# Supplementary material for: Melkersson–Rosenthal Syndrome in Childhood: Report of Three Paediatric Cases and a Review of the Literature
Source: Int J Environ Res Public Health. 2019 Apr 10;16(7):1289. doi: 10.3390/ijerph16071289 (PMC6479526; doi:10.3390/ijerph16071289)
Supplement: Supplementary file 1 [file ijerph-16-01289-s001.pdf]

Laboratory analysis	Instrumental analysis
complete haemogram	otoscopy
serum electrolytes	audiometry
C-reactive protein	electrocardiography
glucose	Cerebrospinal Fluid (CSF) analysis*
creatine phosphokinase	brain Computed Tomography (CT)**
kidney indices	
liver function tests	
bilirubin	
thyroid function tests	
complete autoimmunity panel	

Supplementary Materials Table 1 – List of exams that were performed in all three cases reported in the article. *CSF analysis was performed in Patient #1 and Patient #3. **Brain Computed Tomography was performed in Patient #1 and Patient #2.
